# [*N*,*N*′-Bis(2,4,6-trimethyl­phen­yl)ethane-1,2-diimine-κ^2^ *N*,*N*′]tetra­carbonyl­chromium(0)

**DOI:** 10.1107/S1600536812024786

**Published:** 2012-06-16

**Authors:** Marilé Landman, Roan Fraser, René Pretorius, Rohen Prinsloo, David C. Liles, Petrus H. van Rooyen

**Affiliations:** a Department of Chemistry, University of Pretoria, Private Bag X20, Hatfield 0028, South Africa

## Abstract

The octa­hedral coordination of the Cr^0^ atom in the title compound, [Cr(C_20_H_24_N_2_)(CO)_4_], displays some distortion. This is manifested by an exocyclic torsion angle C(mesitylene)—N—Cr—C(carbon­yl) that deviates by more than 20° from planarity. Another structural feature is the significant distortion from linearity of the Cr—C—O angles of the two carbonyl groups that inter­act with both *ortho-*methyl groups of the two mesitylene rings. The remaining two carbonyl groups overlap with the centres of the mesitylene rings themselves and are linear within <3°.

## Related literature

For the synthesis of similar complexes, see: Baxter & Connor (1995 ▶). The MLCT (metal-to-ligand charge-transfer) band was observed at 570 nm for an analogous complex; see: Ruminski & Wallace (1987 ▶).
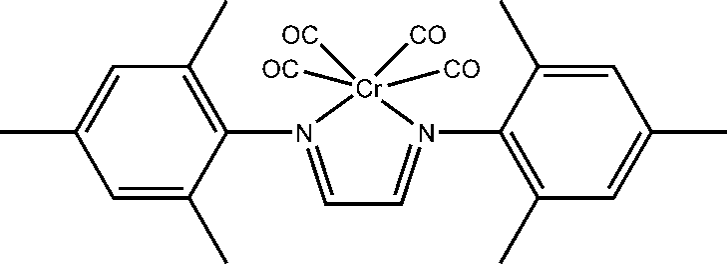


## Experimental

### 

#### Crystal data

[Cr(C_20_H_24_N_2_)(CO)_4_]
*M* *_r_* = 456.45
Monoclinic, 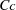

*a* = 19.3119 (18) Å
*b* = 7.5303 (7) Å
*c* = 16.0769 (15) Å
β = 100.912 (2)°
*V* = 2295.7 (4) Å^3^
*Z* = 4
Mo *K*α radiation
μ = 0.53 mm^−1^
*T* = 293 K
0.46 × 0.38 × 0.36 mm

#### Data collection

Adapted Bruker (Siemens) P4 diffractometer
Absorption correction: multi-scan (*SADABS*; Bruker, 2001 ▶) *T*_min_ = 0.676, *T*_max_ = 0.826
5784 measured reflections
2708 independent reflections
2689 reflections with *I* > 2σ(*I*)
*R*_int_ = 0.021

#### Refinement

*R*[*F* ^2^ > 2σ(*F* ^2^)] = 0.029
*wR*(*F* ^2^) = 0.075
*S* = 1.07
2708 reflections
286 parameters
2 restraints
H-atom parameters constrained
Δρ_max_ = 0.19 e Å^−3^
Δρ_min_ = −0.19 e Å^−3^
Absolute structure: Flack (1983 ▶), 652 Friedel pairs
Flack parameter: 0.018 (14)

### 

Data collection: *SMART* (Bruker, 2001 ▶); cell refinement: *SAINT* (Bruker, 2001 ▶); data reduction: *SAINT*; program(s) used to solve structure: *SHELXTL* (Sheldrick, 2008 ▶); program(s) used to refine structure: *SHELXTL* and *SHELXL97* (Sheldrick, 2008 ▶); molecular graphics: *ORTEP-3 for Windows* (Farrugia, 1997 ▶), *POV-RAY* (Cason, 2004 ▶) and *Mercury* (Macrae *et al.*, 2008 ▶); software used to prepare material for publication: *SHELXL97* and *PLATON* (Spek, 2009 ▶).

## Supplementary Material

Crystal structure: contains datablock(s) I, global. DOI: 10.1107/S1600536812024786/wm2632sup1.cif

Structure factors: contains datablock(s) I. DOI: 10.1107/S1600536812024786/wm2632Isup2.hkl

Additional supplementary materials:  crystallographic information; 3D view; checkCIF report

## Figures and Tables

**Table:** 

Cr1—C2	1.860 (3)
Cr1—C1	1.862 (2)
Cr1—C4	1.890 (3)
Cr1—C3	1.897 (3)
Cr1—N2	2.0740 (19)
Cr1—N1	2.0756 (18)

**Table:** 

O1—C1—Cr1	177.1 (2)
O2—C2—Cr1	179.8 (3)
O3—C3—Cr1	172.0 (2)
O4—C4—Cr1	170.1 (3)

**Table:** 

C1—Cr1—N2—C21	−21.80 (19)

**Fig. 1.**
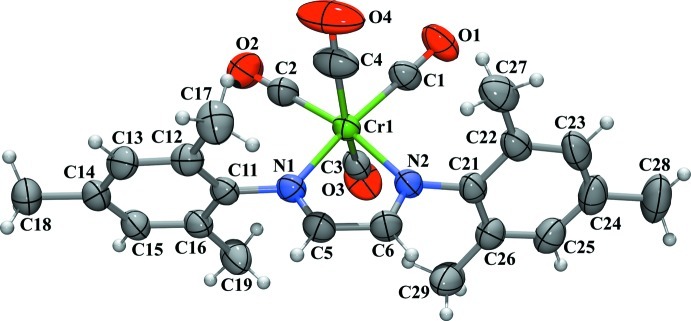
View of the asymmetric unit of (I). Displacement ellipsoids are shown at the 50% probability level.

## supplementary crystallographic information

### Comment

Cr(CO)_4_(*N*,*N*'-dimesitylethylenediimine) or [Cr(CO)_4_(C_20_H_24_N_2_)], (I), was formed as a product in the microwave-assisted reaction of chromium hexacarbonyl with *N*,*N*'-(ethane-1,2-diylidene)bis(2,4,6-trimethylaniline) in dichloromethane as solvent. The intensely-coloured blue-black target complex was formed almost quantitatively with no side products. The complex was characterized using X-ray diffraction, NMR, IR and UV spectroscopy.

The synthesis of similar complexes, using disubstituted 2,2'-bipyridine compounds as coordinating ligands to study solvatochromism of these and other group 6 metal derivatives, was reported previously (Baxter & Connor, 1995). In the UV spectrum of (I), a stronger MLCT band is observed at 595 nm. The intense colour of the complex is ascribed to this metal-to-ligand transition. For an analogous complex, *viz*. [Cr(CO)_4_(dpp)], dpp = 2,3-bis(2-pyridyl)pyrazine, the MLCT band was observed at 570 nm with CHCl_3_ as solvent (Ruminski & Wallace, 1987). The transition around 300 nm was assigned as a dpp π* →π intraligand transition.

The solid state structure of the title compound revealed that the molecule packs with the mesitylene rings close to parallel to the (*ac*)-plane, with the angle between the mean planes formed by these rings at 19.81 (12)°. The 5-membered (Cr—N—C—C—N) ring is planar and approximately parallel to the (*bc*)-plane. This mean plane is almost perpendicular with the mean planes formed by the mesitylene rings, with the values for these angles being 85.42 (11)° and 76.87 (11)°. The distorted octahedral geometry around the Cr^0^ atom (Fig. 1) is manifested by the exocyclic torsion angle C21—N2—Cr1—C1 with a value of -21.81 (19)°. Another structural feature is the significant distortion from linearity, by 8.0° and 9.8°, of the Cr—C—O bond angles of the two carbonyl groups that interact with the *ortho* methyl groups of the two mesitylene rings. These two Cr—CO bond lengths are similar at 1.890 (3) Å (C4) and 1.897 (3) Å (C3), and the corresponding Cr—C—O bond angles are 170.2 (3)° and 172.0 (2)°. The remaining two carbonyl groups are positioned over the centre of the mesitylene rings and have little steric interaction, and are thus linear within 3°. This results in shorter Cr—C bond lengths of 1.861 (3) Å (C2) and 1.863 (3) Å (C1) with the corresponding Cr—C—O bond angles of 177.1 (2)° and 179.8 (3)°.

The crystal packing is without any other significant features, but the value of 18.5 Å^3^ per non-H atom is indicative of the efficient packing of the molecules in the unit cell.

### Experimental

Cr(CO)_6_ (3 mmol, 0.66 g) and *N*,*N*'-(ethane-1,2-diylidene)bis(2,4,6-trimethylaniline) (3 mmol, 0.60 g) were added to a microwave container and 30 ml of dichloromethane added. The container was sealed and the vessel inserted into the microwave oven. The reaction was left at 700 Watt for 1.5 h. The resulting solution was dark blue in colour. Solvent was removed *in vacuo*. The product was isolated on a silica gel column using 1:1 DCM:hexane as solvent. Recrystallization from the same solvent mixture yielded blue-black crystals. ^1^H NMR (δ, p.p.m.), CDCl_3_: 2.20 (s, 12H, *o*-Me), 2.33 (s, 6H, *p*-Me), 6.99 (s, 4H, *m*-H), 8.16 (s, 2H, N—H); ^13^C NMR (δ, p.p.m.), CDCl_3_: 18.2, 20.8, 128.1, 129.3, 135.7, 151.4, 158.9, 213.6, 224.3. IR (νCO, cm^-1^) KBr pellet: 2001(*s*), 1902(*s*), 1916(*s*), 1866(*s*). UV (λ, nm): 595, 362.

### Refinement

All hydrogen atom positions were obtained from difference Fourier maps but were included in the refinement as riding on the atom to which they are bonded. Isotropic displacement parameters for the hydrogen atoms were set at 1.2 times the equivalent isotropic displacement parameter of the atom to which each hydrogen atom is bonded (1.5 times for the methyl H atoms).

### Crystal data

**Table:**

[Cr(C_20_H_24_N_2_)(CO)_4_]	*F*(000) = 952
*M**_r_* = 456.45	*D*_x_ = 1.321 Mg m^−^^3^
Monoclinic, *C**c*	Mo *K*α radiation, λ = 0.71073 Å
Hall symbol: C -2yc	Cell parameters from 5595 reflections
*a* = 19.3119 (18) Å	θ = 2.7–26.4°
*b* = 7.5303 (7) Å	µ = 0.53 mm^−^^1^
*c* = 16.0769 (15) Å	*T* = 293 K
β = 100.912 (2)°	Tetrahedron, dark-blue
*V* = 2295.7 (4) Å^3^	0.46 × 0.38 × 0.36 mm
*Z* = 4	

### Data collection

**Table:**

Adapted Bruker (Siemens) P4 diffractometer	2708 independent reflections
Radiation source: fine-focus sealed tube	2689 reflections with *I* > 2σ(*I*)
Graphite monochromator	*R*_int_ = 0.021
Detector resolution: 8.3 pixels mm^-1^	θ_max_ = 25.2°, θ_min_ = 2.6°
φ and ω scans	*h* = −23→15
Absorption correction: multi-scan (*SADABS*; Bruker, 2001)	*k* = −8→9
*T*_min_ = 0.676, *T*_max_ = 0.826	*l* = −18→15
5784 measured reflections	

### Refinement

**Table:**

Refinement on *F*^2^	Secondary atom site location: difference Fourier map
Least-squares matrix: full	Hydrogen site location: inferred from neighbouring sites
*R*[*F*^2^ > 2σ(*F*^2^)] = 0.029	H-atom parameters constrained
*wR*(*F*^2^) = 0.075	*w* = 1/[σ^2^(*F*_o_^2^) + (0.0589*P*)^2^ + 0.0184*P*] where *P* = (*F*_o_^2^ + 2*F*_c_^2^)/3
*S* = 1.07	(Δ/σ)_max_ = 0.001
2708 reflections	Δρ_max_ = 0.19 e Å^−^^3^
286 parameters	Δρ_min_ = −0.19 e Å^−^^3^
2 restraints	Absolute structure: Flack (1983), 652 Friedel pairs
Primary atom site location: structure-invariant direct methods	Flack parameter: 0.018 (14)

### Special details

**Table:**

Geometry. All e.s.d.'s (except the e.s.d. in the dihedral angle between two l.s. planes) are estimated using the full covariance matrix. The cell e.s.d.'s are taken into account individually in the estimation of e.s.d.'s in distances, angles and torsion angles; correlations between e.s.d.'s in cell parameters are only used when they are defined by crystal symmetry. An approximate (isotropic) treatment of cell e.s.d.'s is used for estimating e.s.d.'s involving l.s. planes.
Refinement. Refinement of *F*^2^ against ALL reflections. The weighted *R*-factor *wR* and goodness of fit *S* are based on *F*^2^, conventional *R*-factors *R* are based on *F*, with *F* set to zero for negative *F*^2^. The threshold expression of *F*^2^ > σ(*F*^2^) is used only for calculating *R*-factors(gt) *etc*. and is not relevant to the choice of reflections for refinement. *R*-factors based on *F*^2^ are statistically about twice as large as those based on *F*, and *R*- factors based on ALL data will be even larger.

### Fractional atomic coordinates and isotropic or equivalent isotropic displacement parameters (Å^2^)

**Table:**

	*x*	*y*	*z*	*U*_iso_*/*U*_eq_	
Cr1	0.212075 (18)	0.27015 (4)	0.64486 (2)	0.04182 (11)	
C1	0.29172 (13)	0.1426 (3)	0.69481 (17)	0.0519 (5)	
O1	0.34138 (11)	0.0624 (3)	0.72228 (16)	0.0767 (6)	
C2	0.14826 (13)	0.0836 (3)	0.63989 (19)	0.0584 (6)	
O2	0.10907 (13)	−0.0316 (3)	0.6367 (2)	0.0928 (8)	
C3	0.19622 (13)	0.2895 (3)	0.75742 (17)	0.0515 (6)	
O3	0.18859 (14)	0.2804 (3)	0.82656 (15)	0.0765 (6)	
C4	0.23428 (19)	0.1662 (5)	0.54614 (19)	0.0731 (8)	
O4	0.2498 (2)	0.0830 (5)	0.49413 (18)	0.1281 (13)	
N1	0.13881 (10)	0.4434 (2)	0.57702 (11)	0.0453 (4)	
N2	0.26563 (9)	0.5096 (2)	0.64851 (12)	0.0439 (4)	
C5	0.16182 (12)	0.6007 (3)	0.56391 (16)	0.0529 (5)	
H5	0.1345	0.6827	0.5289	0.063*	
C6	0.23174 (13)	0.6397 (3)	0.60674 (16)	0.0520 (5)	
H6	0.2517	0.7517	0.6048	0.062*	
C11	0.06672 (11)	0.4040 (3)	0.53884 (13)	0.0440 (4)	
C12	0.04958 (13)	0.3506 (3)	0.45416 (14)	0.0496 (5)	
C13	−0.02004 (14)	0.3115 (4)	0.42077 (15)	0.0544 (5)	
H13	−0.0319	0.2765	0.3644	0.065*	
C14	−0.07296 (12)	0.3223 (4)	0.46827 (15)	0.0525 (5)	
C15	−0.05452 (12)	0.3769 (4)	0.55176 (15)	0.0540 (5)	
H15	−0.0894	0.3854	0.5843	0.065*	
C16	0.01465 (12)	0.4195 (3)	0.58840 (13)	0.0484 (5)	
C17	0.10406 (15)	0.3377 (5)	0.39831 (17)	0.0701 (7)	
H17A	0.1275	0.2250	0.4070	0.105*	
H17B	0.1380	0.4314	0.4124	0.105*	
H17C	0.0813	0.3486	0.3400	0.105*	
C18	−0.14839 (18)	0.2744 (5)	0.4295 (2)	0.0701 (8)	
H18A	−0.1704	0.3727	0.3967	0.105*	
H18B	−0.1739	0.2474	0.4737	0.105*	
H18C	−0.1487	0.1726	0.3935	0.105*	
C19	0.03138 (15)	0.4836 (5)	0.67837 (17)	0.0683 (8)	
H19A	−0.0117	0.5040	0.6985	0.102*	
H19B	0.0577	0.5923	0.6811	0.102*	
H19C	0.0589	0.3955	0.7131	0.102*	
C21	0.33116 (11)	0.5518 (3)	0.70416 (14)	0.0466 (5)	
C22	0.39584 (13)	0.4965 (3)	0.68431 (18)	0.0540 (5)	
C23	0.45673 (12)	0.5362 (4)	0.7433 (2)	0.0678 (7)	
H23	0.5001	0.5020	0.7312	0.081*	
C24	0.45589 (15)	0.6228 (4)	0.8178 (2)	0.0687 (7)	
C25	0.39169 (15)	0.6775 (4)	0.83411 (19)	0.0634 (6)	
H25	0.3905	0.7385	0.8841	0.076*	
C26	0.32877 (13)	0.6448 (3)	0.77868 (16)	0.0539 (5)	
C27	0.39987 (16)	0.4046 (5)	0.6026 (2)	0.0702 (8)	
H27A	0.4453	0.4252	0.5885	0.105*	
H27B	0.3639	0.4504	0.5583	0.105*	
H27C	0.3930	0.2793	0.6087	0.105*	
C28	0.5228 (2)	0.6584 (6)	0.8816 (3)	0.0993 (13)	
H28A	0.5255	0.5782	0.9286	0.149*	
H28B	0.5224	0.7785	0.9015	0.149*	
H28C	0.5629	0.6410	0.8553	0.149*	
C29	0.26070 (19)	0.7080 (4)	0.8015 (3)	0.0721 (8)	
H29A	0.2231	0.6299	0.7770	0.108*	
H29B	0.2506	0.8261	0.7801	0.108*	
H29C	0.2651	0.7084	0.8620	0.108*	

### Atomic displacement parameters (Å^2^)

**Table:**

	*U*^11^	*U*^22^	*U*^33^	*U*^12^	*U*^13^	*U*^23^
Cr1	0.04006 (17)	0.03597 (17)	0.04822 (18)	0.00392 (16)	0.00531 (11)	0.00317 (16)
C1	0.0515 (12)	0.0419 (12)	0.0636 (13)	0.0048 (10)	0.0140 (10)	0.0105 (10)
O1	0.0578 (11)	0.0699 (13)	0.1017 (15)	0.0235 (10)	0.0131 (10)	0.0264 (12)
C2	0.0509 (13)	0.0436 (13)	0.0790 (16)	0.0050 (11)	0.0078 (11)	−0.0002 (11)
O2	0.0716 (14)	0.0568 (12)	0.151 (2)	−0.0180 (11)	0.0228 (14)	−0.0118 (14)
C3	0.0418 (11)	0.0514 (14)	0.0601 (16)	0.0053 (10)	0.0066 (10)	0.0071 (10)
O3	0.0736 (15)	0.1005 (17)	0.0578 (13)	0.0106 (11)	0.0187 (10)	0.0143 (10)
C4	0.094 (2)	0.0626 (17)	0.0631 (16)	0.0289 (16)	0.0159 (14)	0.0080 (13)
O4	0.201 (3)	0.114 (2)	0.0746 (15)	0.079 (2)	0.0382 (19)	−0.0077 (15)
N1	0.0472 (9)	0.0409 (9)	0.0457 (9)	0.0068 (7)	0.0033 (7)	0.0012 (7)
N2	0.0392 (8)	0.0408 (9)	0.0522 (9)	0.0017 (7)	0.0100 (7)	0.0032 (7)
C5	0.0520 (12)	0.0423 (12)	0.0605 (13)	0.0081 (9)	0.0007 (9)	0.0082 (9)
C6	0.0521 (12)	0.0385 (11)	0.0641 (13)	−0.0009 (9)	0.0075 (10)	0.0070 (10)
C11	0.0448 (11)	0.0395 (11)	0.0444 (10)	0.0074 (8)	−0.0002 (8)	0.0023 (8)
C12	0.0565 (12)	0.0492 (13)	0.0423 (10)	0.0042 (10)	0.0075 (9)	0.0019 (9)
C13	0.0577 (13)	0.0592 (12)	0.0415 (11)	0.0062 (12)	−0.0030 (9)	−0.0058 (10)
C14	0.0462 (12)	0.0503 (12)	0.0559 (13)	0.0070 (11)	−0.0029 (9)	−0.0019 (10)
C15	0.0446 (11)	0.0595 (13)	0.0559 (13)	0.0095 (10)	0.0047 (9)	−0.0060 (10)
C16	0.0471 (11)	0.0506 (13)	0.0444 (11)	0.0099 (9)	0.0010 (8)	−0.0047 (9)
C17	0.0644 (16)	0.099 (2)	0.0479 (13)	−0.0018 (16)	0.0138 (11)	−0.0036 (14)
C18	0.0518 (16)	0.080 (2)	0.0725 (18)	0.0039 (12)	−0.0041 (13)	−0.0128 (14)
C19	0.0547 (12)	0.094 (2)	0.0524 (13)	0.0149 (14)	0.0005 (10)	−0.0182 (13)
C21	0.0392 (10)	0.0421 (11)	0.0580 (12)	−0.0022 (8)	0.0078 (9)	0.0070 (9)
C22	0.0453 (11)	0.0480 (12)	0.0707 (14)	0.0018 (9)	0.0162 (10)	0.0114 (10)
C23	0.0383 (12)	0.0670 (16)	0.098 (2)	−0.0027 (11)	0.0126 (12)	0.0189 (15)
C24	0.0564 (14)	0.0599 (15)	0.0838 (19)	−0.0161 (12)	−0.0019 (12)	0.0153 (14)
C25	0.0675 (16)	0.0540 (13)	0.0657 (15)	−0.0134 (13)	0.0046 (12)	0.0007 (12)
C26	0.0506 (12)	0.0477 (13)	0.0640 (14)	−0.0078 (10)	0.0127 (10)	0.0000 (10)
C27	0.0645 (16)	0.0698 (18)	0.0823 (18)	0.0057 (14)	0.0293 (13)	0.0070 (15)
C28	0.0674 (19)	0.093 (3)	0.121 (3)	−0.0246 (19)	−0.0228 (19)	0.014 (2)
C29	0.0657 (17)	0.0710 (17)	0.085 (2)	−0.0084 (14)	0.0280 (15)	−0.0213 (15)

### Geometric parameters (Å, º)

**Table:**

Cr1—C2	1.860 (3)	C17—H17B	0.9600
Cr1—C1	1.862 (2)	C17—H17C	0.9600
Cr1—C4	1.890 (3)	C18—H18A	0.9600
Cr1—C3	1.897 (3)	C18—H18B	0.9600
Cr1—N2	2.0740 (19)	C18—H18C	0.9600
Cr1—N1	2.0756 (18)	C19—H19A	0.9600
C1—O1	1.148 (3)	C19—H19B	0.9600
C2—O2	1.146 (3)	C19—H19C	0.9600
C3—O3	1.151 (4)	C21—C26	1.396 (3)
C4—O4	1.130 (4)	C21—C22	1.409 (3)
N1—C5	1.296 (3)	C22—C23	1.396 (4)
N1—C11	1.442 (3)	C22—C27	1.500 (4)
N2—C6	1.294 (3)	C23—C24	1.368 (5)
N2—C21	1.440 (3)	C23—H23	0.9300
C5—C6	1.425 (3)	C24—C25	1.378 (4)
C5—H5	0.9300	C24—C28	1.514 (4)
C6—H6	0.9300	C25—C26	1.386 (4)
C11—C12	1.398 (3)	C25—H25	0.9300
C11—C16	1.401 (3)	C26—C29	1.507 (4)
C12—C13	1.382 (4)	C27—H27A	0.9600
C12—C17	1.510 (3)	C27—H27B	0.9600
C13—C14	1.389 (4)	C27—H27C	0.9600
C13—H13	0.9300	C28—H28A	0.9600
C14—C15	1.384 (3)	C28—H28B	0.9600
C14—C18	1.516 (4)	C28—H28C	0.9600
C15—C16	1.391 (3)	C29—H29A	0.9600
C15—H15	0.9300	C29—H29B	0.9600
C16—C19	1.501 (3)	C29—H29C	0.9600
C17—H17A	0.9600		
			
C2—Cr1—C1	96.63 (12)	C12—C17—H17C	109.5
C2—Cr1—C4	84.56 (15)	H17A—C17—H17C	109.5
C1—Cr1—C4	81.28 (13)	H17B—C17—H17C	109.5
C2—Cr1—C3	82.74 (12)	C14—C18—H18A	109.5
C1—Cr1—C3	84.11 (11)	C14—C18—H18B	109.5
C4—Cr1—C3	159.37 (13)	H18A—C18—H18B	109.5
C2—Cr1—N2	168.64 (9)	C14—C18—H18C	109.5
C1—Cr1—N2	93.98 (9)	H18A—C18—H18C	109.5
C4—Cr1—N2	101.09 (13)	H18B—C18—H18C	109.5
C3—Cr1—N2	94.36 (10)	C16—C19—H19A	109.5
C2—Cr1—N1	94.11 (9)	C16—C19—H19B	109.5
C1—Cr1—N1	167.48 (9)	H19A—C19—H19B	109.5
C4—Cr1—N1	93.40 (10)	C16—C19—H19C	109.5
C3—Cr1—N1	103.65 (9)	H19A—C19—H19C	109.5
N2—Cr1—N1	75.83 (7)	H19B—C19—H19C	109.5
O1—C1—Cr1	177.1 (2)	C26—C21—C22	121.1 (2)
O2—C2—Cr1	179.8 (3)	C26—C21—N2	118.43 (19)
O3—C3—Cr1	172.0 (2)	C22—C21—N2	120.4 (2)
O4—C4—Cr1	170.1 (3)	C23—C22—C21	116.9 (3)
C5—N1—C11	116.85 (18)	C23—C22—C27	121.1 (2)
C5—N1—Cr1	116.04 (15)	C21—C22—C27	122.0 (2)
C11—N1—Cr1	126.99 (14)	C24—C23—C22	123.3 (2)
C6—N2—C21	117.0 (2)	C24—C23—H23	118.4
C6—N2—Cr1	116.32 (16)	C22—C23—H23	118.4
C21—N2—Cr1	125.75 (14)	C23—C24—C25	118.0 (3)
N1—C5—C6	115.6 (2)	C23—C24—C28	121.9 (3)
N1—C5—H5	122.2	C25—C24—C28	120.1 (3)
C6—C5—H5	122.2	C24—C25—C26	122.4 (3)
N2—C6—C5	115.7 (2)	C24—C25—H25	118.8
N2—C6—H6	122.1	C26—C25—H25	118.8
C5—C6—H6	122.1	C25—C26—C21	118.3 (2)
C12—C11—C16	120.98 (19)	C25—C26—C29	119.1 (3)
C12—C11—N1	120.35 (19)	C21—C26—C29	122.6 (2)
C16—C11—N1	118.67 (18)	C22—C27—H27A	109.5
C13—C12—C11	118.2 (2)	C22—C27—H27B	109.5
C13—C12—C17	119.4 (2)	H27A—C27—H27B	109.5
C11—C12—C17	122.3 (2)	C22—C27—H27C	109.5
C12—C13—C14	122.5 (2)	H27A—C27—H27C	109.5
C12—C13—H13	118.8	H27B—C27—H27C	109.5
C14—C13—H13	118.8	C24—C28—H28A	109.5
C15—C14—C13	118.0 (2)	C24—C28—H28B	109.5
C15—C14—C18	121.4 (2)	H28A—C28—H28B	109.5
C13—C14—C18	120.6 (2)	C24—C28—H28C	109.5
C14—C15—C16	121.9 (2)	H28A—C28—H28C	109.5
C14—C15—H15	119.0	H28B—C28—H28C	109.5
C16—C15—H15	119.0	C26—C29—H29A	109.5
C15—C16—C11	118.35 (19)	C26—C29—H29B	109.5
C15—C16—C19	119.8 (2)	H29A—C29—H29B	109.5
C11—C16—C19	121.9 (2)	C26—C29—H29C	109.5
C12—C17—H17A	109.5	H29A—C29—H29C	109.5
C12—C17—H17B	109.5	H29B—C29—H29C	109.5
H17A—C17—H17B	109.5		
			
C2—Cr1—N1—C5	−179.67 (19)	C11—C12—C13—C14	−0.4 (4)
C1—Cr1—N1—C5	−30.5 (6)	C17—C12—C13—C14	−179.3 (3)
C4—Cr1—N1—C5	−94.9 (2)	C12—C13—C14—C15	0.9 (4)
C3—Cr1—N1—C5	96.82 (18)	C12—C13—C14—C18	−178.7 (3)
N2—Cr1—N1—C5	5.70 (17)	C13—C14—C15—C16	−0.3 (4)
C2—Cr1—N1—C11	−3.74 (19)	C18—C14—C15—C16	179.3 (3)
C1—Cr1—N1—C11	145.4 (5)	C14—C15—C16—C11	−0.8 (4)
C4—Cr1—N1—C11	81.0 (2)	C14—C15—C16—C19	178.0 (3)
C3—Cr1—N1—C11	−87.25 (18)	C12—C11—C16—C15	1.3 (4)
N2—Cr1—N1—C11	−178.36 (18)	N1—C11—C16—C15	−178.4 (2)
C2—Cr1—N2—C6	−31.3 (6)	C12—C11—C16—C19	−177.5 (2)
C1—Cr1—N2—C6	169.63 (18)	N1—C11—C16—C19	2.8 (4)
C4—Cr1—N2—C6	87.7 (2)	C6—N2—C21—C26	71.7 (3)
C3—Cr1—N2—C6	−105.99 (18)	Cr1—N2—C21—C26	−96.8 (2)
N1—Cr1—N2—C6	−3.00 (17)	C6—N2—C21—C22	−109.7 (3)
C2—Cr1—N2—C21	137.3 (5)	Cr1—N2—C21—C22	81.8 (2)
C1—Cr1—N2—C21	−21.80 (19)	C26—C21—C22—C23	1.2 (4)
C4—Cr1—N2—C21	−103.68 (19)	N2—C21—C22—C23	−177.4 (2)
C3—Cr1—N2—C21	62.59 (18)	C26—C21—C22—C27	−176.9 (2)
N1—Cr1—N2—C21	165.58 (18)	N2—C21—C22—C27	4.6 (4)
C11—N1—C5—C6	176.19 (19)	C21—C22—C23—C24	0.6 (4)
Cr1—N1—C5—C6	−7.5 (3)	C27—C22—C23—C24	178.7 (3)
C21—N2—C6—C5	−169.4 (2)	C22—C23—C24—C25	−1.8 (4)
Cr1—N2—C6—C5	0.2 (3)	C22—C23—C24—C28	178.0 (3)
N1—C5—C6—N2	4.8 (3)	C23—C24—C25—C26	1.2 (4)
C5—N1—C11—C12	82.6 (3)	C28—C24—C25—C26	−178.6 (3)
Cr1—N1—C11—C12	−93.3 (2)	C24—C25—C26—C21	0.5 (4)
C5—N1—C11—C16	−97.7 (2)	C24—C25—C26—C29	179.6 (3)
Cr1—N1—C11—C16	86.4 (2)	C22—C21—C26—C25	−1.7 (4)
C16—C11—C12—C13	−0.7 (4)	N2—C21—C26—C25	176.8 (2)
N1—C11—C12—C13	178.9 (2)	C22—C21—C26—C29	179.2 (3)
C16—C11—C12—C17	178.2 (3)	N2—C21—C26—C29	−2.2 (4)
N1—C11—C12—C17	−2.2 (4)		
